# The effect of temperature on male mating signals and female choice in the red mason bee, *Osmia bicornis* (L.)

**DOI:** 10.1002/ece3.3331

**Published:** 2017-09-23

**Authors:** Taina Conrad, Carina Stöcker, Manfred Ayasse

**Affiliations:** ^1^ Institute of Evolutionary Ecology and Conservation Genomics University of Ulm Ulm Germany

**Keywords:** climate change, insects, pheromones, sexual selection, vibrational communication

## Abstract

Climate change and the resulting changes in air temperature are known to have a major influence on most animals, especially poikilothermic insects, because they depend on the high enough temperatures to function. Previous studies have shown that various signals can be affected by changes in temperature. However, research into the effect of temperature on mating signals and subsequently communication between mates and on female choice is still rare. In the red mason bee, *Osmia bicornis*, which emerges early in spring and is therefore subject to extensive temperature changes, females choose suitable males based on, among other criteria, their thorax vibrations and odor. So far there has been no research into the effect of temperature changes on these signals. We therefore investigated whether the environmental temperature has an influence on the male's mating signals by measuring vibrations using a laser vibrometer and collecting male and female odor at different temperatures. Furthermore, we performed behavioral experiments in order to show whether there is an effect of temperature‐induced changes and female choice. Our results showed that rejected males differed in their vibrations between the two temperature settings but accepted ones did not. Temperature changes therefore seem to have a stronger effect on those males that are rejected by the females, whereas the accepted males are the ones that can produce desirable signals despite temperature fluctuations. Furthermore, we found that the differences in odor profiles were greater between temperature settings than between the sexes and that females change their preference for odor with temperature. We conclude that temperature strongly influences the male mating signals and therefore may have a major impact on sexual selection in this species. This is an important aspect to consider, not only in future studies on mating behavior, but also in view of our ever raising temperatures.

## 1 INTRODUCTION

Climate change has become a reality we are facing (Parmesan & Yohe, 2003), and in recent years, temperature changes and their effect on natural systems have moved into focus. Many plant and animal species from various different taxonomical groups are known to be affected in one way or another by environmental changes (Grace & Shaw, 2004; Greenfield, 2002; Kearney, Shine, & Porter, 2009; Pörtner & Knust, 2007; Root et al., 2003; Sentis, Ramon‐Portugal, Brodeur, & Hemptinne, 2015). Temperature fluctuations are of particular importance because almost every organism has to deal with smaller or greater differences in temperature during its life. This is especially true for poikilothermic animals, which depend on a certain environmental temperature in order to function. The surrounding temperature not only influences the body temperature of an individual, but also can affect their communication systems, as is the case in electric fish (Feng, 1976), fireflies (Carlson, Copeland, Raderman, Bulloch, & Andrew, 1976), anurans (Gayou, 1984; Zweifel, 1968), and orthoptera (Pires & Hoy, 1992; Walker, 1975).However, research, especially in insects, is still rare and so far very biased (Andrew et al., 2013). A disproportionate amount of research has focused on orders with lesser species richness like the lepidoptera, diptera, or orthoptera while in coleoptera, hymenoptera, and hemiptera, there are fewer studies relative to their overwhelming species richness (Andrew et al., 2013). Additionally, most studies focus on changes in distribution or range shift and very few on behavior or life history traits (Andrew et al., 2013). Especially studies on the effect of temperature on communication signals in animals and their consequence are scarce.

Vibrational signals are widespread throughout the animal kingdom, can be found in almost every class of animal (Cocroft & Rodriguez, 2005; Hill, 2008) and are estimated to be found in 92% of the described insect species (Cocroft & Rodriguez, 2005). Vibrational communication is in fact probably one of the most primary signaling channels, even predating the ear mechanism (Hill, 2008), and is used in various different functions, as in predator–prey interactions (DeVries, 1990; Hill, 2008), foraging (Hrncir, Jarau, Zucchi, & Barth, 2004), mating (DeLuca & Morris, 1998), and many others (Hill, 2008). In many cases, female choice is based on these vibrational signals, for example, in planthoppers (Heady & Denno, 1991) and treehoppers (Rodríguez, Laura Sullivan, & Cocroft, 2004). In the treehopper *Enchenopa binotata*, for example, the female chooses its male based on substrate‐borne vibrations. Various attributes of the male signal are responsible for the female choice, for example, base frequency, length of the whine section, pulse rate, and number of pulses (Rodríguez et al., 2004). There are also many reports of intraspecific differences in these vibrational signals between populations and their influence in mate choice (Claridge & de Vrjer 1993; Čokl, Virant‐Doberlet, & Stritih, 2000; Gillham, 1992; Miklas, Čokl, Renou, & Virant‐Doberlet, 2003; Ryan, C̊okl, & Walter, 1996).

Although there are studies on the effect the temperature changes can have on acoustic signals in insects, most of them focus on the orthoptera, which have specialized sound producing organs. In anurans and crickets, the studies show that temporal parameters of the song are particularly affected by temperature (Gayou, 1984; Pires & Hoy, 1992). An increase in air temperature usually correlates with an increase in pulse rate (Gayou, 1984; Greenfield, 2002). There is evidence that an increase in temperature might also lead to changes in the main frequency of the call; although the effect has mostly been found to be quite small, as is the case in *Hyla versicolor* where frequency increased only slightly with temperature (Gayou, 1984). In theory, though, higher body temperatures can elevate the muscle contraction rate, which means faster oscillation and therefore higher frequencies (Greenfield, 2002). Research into the effects of temperature on vibrational communication in insects is, to our knowledge, so far lacking completely.

Pheromones located on the cuticle surface play an important role in insect mating behavior and have previously been studied in several bees (Ayasse, Paxton, & Tengö, 2001). Female pheromones are used to attract males, to identify receptive females, and to elicit territorial behavior and courtship behavior in males (Ayasse, Engels, & Francke, 1999; Ayasse et al., 2001; Krieger et al., 2006). Less information exists on the function of male sex pheromones. In megachilid and xylocopine bees, epidermal odor glands were found in the foreleg basitarsi of males (Gerhardt & Mudry, 1980). During copulation, the openings of these glands come into close contact with the antennae of the female. Whether the pheromones of these males increase the likelihood that the females will mate was not experimentally proven (Gerhardt & Mudry, 1980). In many bees, secretions produced by male mandibular glands or labial glands are used to mark spots along male flight paths (Ayasse & Jarau, 2014; Tengo & Kullenberg, 1979; Vinson et al., 1982) or sometimes in a female's choice for a suitable male (Ayasse et al., 2001).

The effect of temperature on odor has gotten more attention than vibrational communication in the past. In insects, cuticular hydrocarbons have primarily evolved to serve as a barrier against water loss (Edney, 1967; Hadley, 1986). Over time, a secondary function as communication signals evolved (Blomquist & Bagnères, 2010). Most insects have to deal with smaller or greater changes in temperature throughout their life and have consequently developed various adaptations to prevent water loss (Prosser & Nelson, 1981; Wingfield, 2003). Previous studies were able to show that these adaptations include changes in the odor profile on the cuticle (Noorman & Den Otter, 2002). To adapt to higher temperatures, most insects change their odor profile toward higher n‐alkanes with lower melting points (Gibbs, Louie, & Ayala, 1998). If part of the odor profile serves in intraspecific communication, however, one would expect changes like that to be fatal if communication, for example through pheromones, is disrupted. Therefore research into changes of infochemical‐mediated interactions and subsequent changes in behavior due to temperature fluctuations remains rare (Sentis et al., 2015).

**Figure 1:**
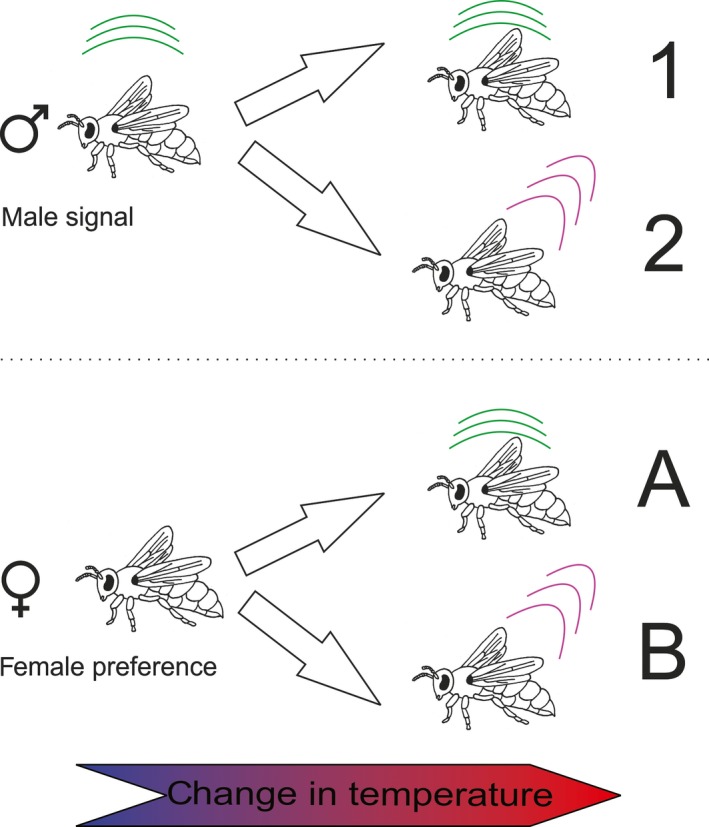
Possible changes in male signals and female preference due to changes in temperature. There are two possible ways a male signal can react to temperature changes: either the signal stays the same (1) or the signal changes (2). Female preference can also either stay the same (A) or females can adapt to a change in temperature with a change in preference for a, now changed, male signal (B)

In cases of female choice, there are generally two ways females can “deal” with a change in the male signal due to temperature changes. One way is to adapt to the changes and interpret the signal accordingly. This parallel change in male signal and female preference due to a change in temperature is called “temperature coupling” (Gerhardt, 1978), and it is still being discussed if this is an adaptive function or coincidental (Greenfield & Medlock, 2007; Ritchie, Saarikettu, Livingstone, & Hoikkala, 2001). There are various examples of this, especially in insects and frogs (Doherty, 1985; Gerhardt, 1978; Pires & Hoy, 1992; Randall, Eckert, Burggren, & French, 1997). In the katydid *Neoconocephalus triops,* for example, female preference changed with temperature to be matched up with the male amplitude modulation rates (Beckers & Schul, 2008). Another possibility is that the female preference stays the same, regardless of temperature. In this case, those males whose signals are less affected by temperature would have an advantage (Figure 1). Examples for this are rare, although *Drosophila montana* and *Chortippus biguttulus* both show no temperature coupling (von Helversen & von Helversen, 1981; Ritchie et al., 2001), and in *Hyla versicolor,* the female preference changes with temperature but the male signal hardly does (Gerhardt & Mudry, 1980). Both possibilities of influence of temperature on male signal and female preference are therefore plausible and need to be investigated.

**Figure 2:**
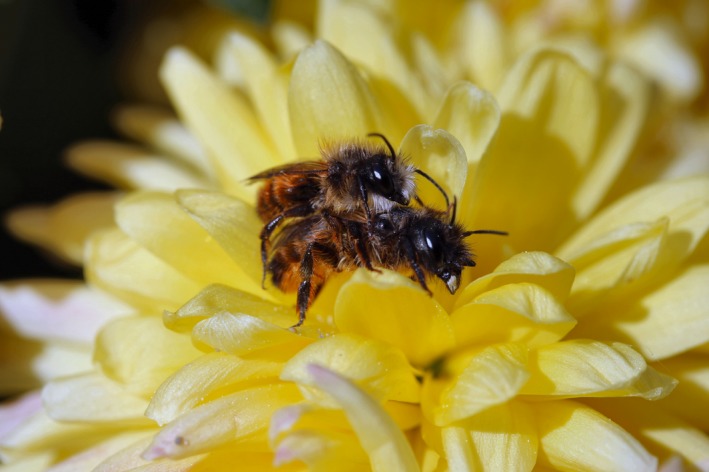
A couple of *Osmia bicornis* with the male sitting on top of the female in a precopulatory embrace

The red mason bee, *Osmia bicornis* (Figure 2), is a solitary univoltine bee, which is widespread throughout Europe and an important pollinator of many plants, including crop plants like apple trees (O'Toole, 2000). It is easily reared in trap nests and due to the “pollinator crisis” and a decline of honeybee populations, it is becoming more and more important as an alternative pollinator (O'Toole, 2000). It emerges early in spring at temperatures around 15°C. However, given the fluctuating weather conditions in the month of April in central Europe, they can sometimes experience temperatures up to 25°C (Seidelmann, 1991).

After a female emerges and leaves the nest, patrolling males, attracted by a female sex pheromone (Rosner, 1994), pounce on her and try to copulate. Once a male has mounted a female, it shows an elaborate mating behavior, during which the male sits on the female's back embracing her with his hindlegs. He then strokes his antennae and front legs over her antennae and in front of her eyes while vibrating his thorax to emit an audible buzzing sound (Seidelmann, 1995). A female most likely detects a male's odor during precopulation when the male strokes the female's antennae with its own, or when the female contacts the male body surface with the chemoreceptors of her own antennae (Conrad, Paxton, Barth, Francke, & Ayasse, 2010). It is so far unknown if the chemicals are contact pheromones or if they are released, but the antennae of male and female do not seem to touch during precopulation (Conrad, personal observation). The female then uses the vibrational signals as well as chemical signals emitted during this behavior in her choice for a suitable mating partner (Conrad & Ayasse, 2015; Conrad et al., 2010).

Temperature might affect the mating behavior in this bee intensely. Males’ signals may change with air temperature, influencing the female choice in the precopulatory behavior. The resulting change in sexual selection could then in turn lead to an unknown change in the population. However, so far research into the effect of temperature changes on these bees has focused on the influence on overwintering *Osmia* (Bosch & Kemop, 2004; Sgolastra et al., 2011; Wasielewski, Wojciechowicz, Giejdasz, & Krishnan, 2011).

In this study, we therefore wanted to answer the question whether the environmental temperature influences the male's vibrations and odor bouquets and whether this has an influence on female choice. We conducted mating experiments under controlled temperature settings with a high and a low‐temperature setup in order to compare the vibrational signals produced by the males. Additionally, we analyzed the odor profile of males and females from both temperatures to see whether it was affected by the change.

## 2 METHODS

### 2.1 Animals

We used bees from a population of *O. bicornis cornigera,* reared at the University of Ulm in trap nests (50 cm × 20 cm × 23 cm). *Osmia bicornis* cocoons containing teneral adults are overwintered in the field, removed from the trap nests in January, and then stored in a climate chamber at 5°C until they are required for experiments. Remaining bees not used in experiments are emerged next to the trap nests to ensure next year's adults. This population is mixed, consisting of bees from Ulm, Vienna, and Gattersleben—locations with different climatic conditions. However, they have been in Ulm for over 20 years now, so we believe any differences in adaptations to have disappeared. The cocoons were then moved into a further climate chamber with two temperature settings (17 and 22°C), where they emerged in separate flight cages. Additional cocoons were added during the experiment so that a sufficient number of bees were available at all times. After emergence (usually after about 7 days), they were provided ad libitum with a 50% (w/w) sugar solution of API‐Invert (72.7%; Südzucker AG, Rain, Germany; 1 g citric acid and 3 g potassium sorbate were added per liter API‐Invert solution).

The low‐temperature experiments were conducted at 17–21°C, and the high‐temperature experiments were conducted at 22–26°C. There was a 3‐day acclimation period between the two settings. Male bees were used from emergence until they died or engaged with a female, and female bees were used straight after emergence and up to 1 day. Temperatures varied across 4°C because of the heat introduced by the two observers in the chamber. To ensure that the broad temperature setting did not lead to some sort of sorting of accepted versus rejected males, the temperature for each individual experiment was recorded. There was no sorting that could be observed (Pearson's correlation, *r* = −.181, *p* < .05).

### 2.2 Laser vibrometry

**Figure 3:**
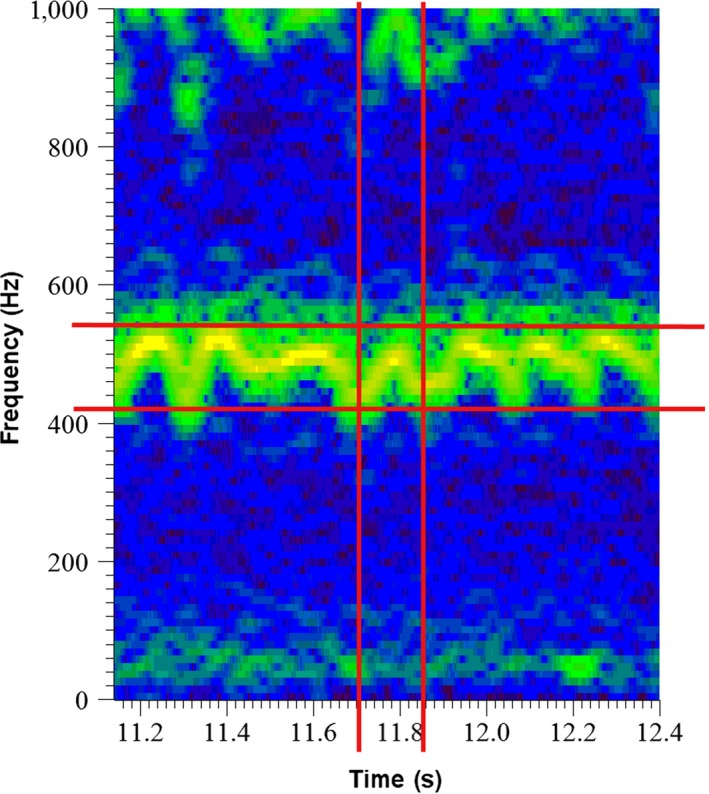
Measurements taken from the vibrations of a male bee. 1 = modulation range, 2 = pulse duration

Thorax vibrations were recorded in the climate chamber between 10 April and 25 May 2012 to compare the effect of two different sets of temperatures on thorax vibrations. Male and female bees used for the recordings had emerged in separate flight cages, and one female at a time was introduced into a flight cage with about 40 males. Once a mating pair had become established in precopulatory embrace (one male sitting on a female and the other males retreating), the pair was taken out of the flight cage and put on a white table. Afterward, we registered if a male was accepted for copulation or rejected by a female. The thoracic vibrations produced by males during the precopulatory phase were recorded with a laser vibrometer (PolytecPDV‐100; Waldbronn, Germany) connected to a desktop computer using a 32‐bit sound card and Soundforge 9.0 software (SonicFoundry, Madison, WI, USA) at a sampling rate of 44.1 kHz. The files were later analyzed using Spike 2.0 (Cambridge Electronic Design). All males were marked with a white spot on their thorax to enhance the reflection of the laser beam. In order to evaluate the data, we compared frequency, pulse duration, and modulation parameters (Figure 3).

Each observed mating pair was afterward frozen in liquid nitrogen and kept at −4°C till further analysis. We were able to measure 38 mating pairs at low temperatures and 35 at high temperatures. We recorded and compared the vibrations of males that were either accepted (permitted by the female to copulate) or rejected by the female. Females occasionally moved during the recordings, and the angle between the laser and the bees changed. Hence, we were unable to compare the signal amplitudes of the different males.

### 2.3 Chemical analysis

As previous studies have shown that females appear to use the antennal odor of the males in their choice (Conrad et al., 2010; Conrad T., unpublished data), we used antennae of male and female bees from the two different temperature settings for chemical analyses. In order to obtain volatiles of the cuticular surface, both antennae of a bee were cut off and extracted in a vial with 100 μl of pentane (99%, Sigma Aldrich Chemie GmbH) at 5°C for 24 hr and then removed. The pentane was evaporated under a nitrogen stream to a volume of 10 μl and an internal standard of 1 μg undecan (stock solution 100 μg/ml) was added.

To determine the cuticular odor profile of individual bees, samples were analyzed using a gas chromatograph (GC; HP 5890, Series II, Hewlett Packard, Palo Alto, CA) equipped with an FID (flame ionization detector) and a nonpolar DB‐5 column (30 m × 0.25 mm i.d. × 50 μm film, J&W) and hydrogen (2 ml/min) as carrier gas. One microlitre of the sample was injected splitless at an initial oven temperature of 50°C. After 1 min, the splitting valve was opened, and the temperature then increased by 10°C/min until it reached 310°C, where it was kept constant for 50 min. In order to ensure consistency in the analyses, a GC run with a synthetic alkane standard mixture was regularly performed. Structure elucidation of individual compounds was performed with an HP 6890 gas chromatograph (Hewlett Packard) connected to a mass selective detector (GCMS; Quadrupol 5972, Agilent, Santa Clara, CA, USA). The temperature program was the same as described above. Helium was used as the carrier gas (1.5 ml/min constant flow). Based on previous work (Ayasse et al., 2001; Conrad et al., 2010; Ibarra, 2000; Rosner, 1994), structure assignments were carried out by comparison of mass spectra and retention times of natural products with corresponding data from synthetic reference samples, using the NIST database and a database of the Institute of Evolutionary Ecology and Conservation Genomics at the University of Ulm. Peak identities between different runs were confirmed by GC/MS.

### 2.4 Statistics

For statistical analysis of the data, we used SigmaStat 3.1 (Systat Software, Chicago, IL, USA), Sigma Plot 9 (Systat Software) and SPSS 13.0 (SPSS Inc., München, Germany). All data were checked for deviation from a normal distribution using a Kolmogorov–Smirnov test. Because the laser vibrometry data were not always normally distributed, the data at low and high‐temperature settings were compared using Mann–Whitney *U* tests. The normally distributed data were compared using a *t* test.

Relative amounts of compounds found on the surface of the males’ antennae were used in nonparametric multivariate analyses to test for temperature related differences in odor bouquets. We performed a nonmetric multidimensional scaling analysis (NMMDS) using the program PAST (Ryan, Harper, & Whalley, 1995), followed by a nonparametric multivariate statistical analysis (one‐way analysis of similarities (ANOSIM)) to test for significance of differences between the odor bouquets (calculated as Bray–Curtis distances) of males and females collected at different temperatures. After 10,000 permutations, the resulting *R* values were used as a measure of dissimilarity (*R* = 0 means two groups are the same, *R* = 1 means they are completely differentiated). Significance was assessed after sequential Bonferroni adjustment of *p* values. A subsequent SIMPER analysis was used to identify the substances responsible for the differences between the different odor profiles.

## 3 RESULTS

### 3.1 Vibrations

**Figure 4:**
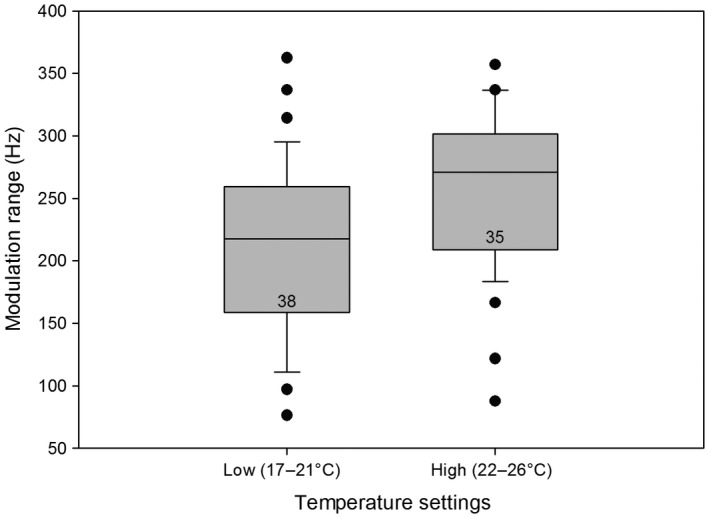
Comparison of modulation ranges during different temperature settings. The medians, quartiles, outliers (circles), and sample sizes (numbers) are shown. There was a significantly higher modulation range at higher temperatures (*t* test, *p* < .05)

Our comparison of the main frequency, modulation range, and pulse duration of the vibrations of males kept at low or high temperatures showed significant differences in the modulation range (Figure 4), but not in pulse duration or the main frequency (*t* test, *p* < .05; Mann–Whitney *U* test, *p* < .005).

**Figure 5:**
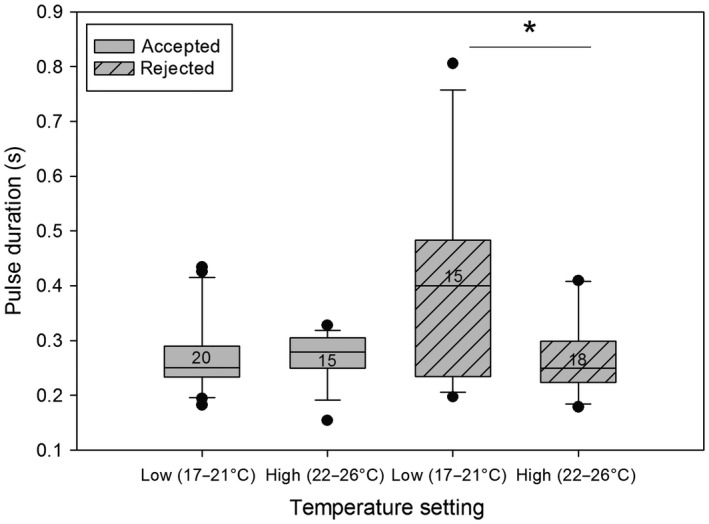
Comparison of the pulse duration during different temperature settings between accepted and rejected males. The medians, quartiles, outliers (circles), and sample sizes (numbers) are shown. Significant differences are marked by an asterisk (*Mann–Whitney *U* test, *p* < .05). Rejected males showed significantly longer pulse durations at lower temperature

**Figure 6:**
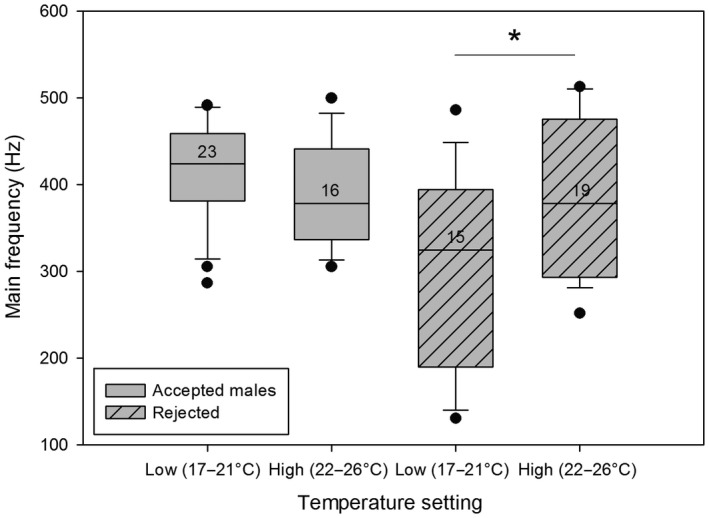
Comparison of the main frequency during different temperature settings between accepted and rejected males. The medians, quartiles, outliers (circles), and sample sizes (numbers) are shown. Significant differences are marked by an asterisk (*t test, *p* < .05). Rejected males showed significantly lower main frequencies at lower temperature

A closer look at which males were accepted or rejected by the females in the different temperature settings revealed a significant difference between the rejected males but not the accepted ones for the time of the pulse duration (Figure 5) and the main frequency (Figure 6; Mann–Whitney *U* test, *p* < .05).

Rejected males showed longer pulse durations and a lower main frequency at lower temperatures. It is therefore the rejected males that show variations in their signals, whereas the signals of the accepted males do not differ between temperature settings.

### 3.2 Chemical analysis

We were able to register 27 compounds on the male antennae, mainly alkanes and alkenes with chain lengths between 20 and 32 carbon atoms and double bond position 7, 9, or 11. Additionally, we found two sterols (Fig. S1 and Table 1).

**Table 1:** List of compounds assigned in the extracts of male *Osmia bicornis*. Numbers correspond to the peaks in Fig. S1. We used 28 compounds in the analysis and were able to identify 23 by using GC/Ms or reference runs with pure substances

No.	Compound name	No.	Compound name
1	Icosane	15	(Z)‐7‐Heptacosene
2	Heneicosane	16	Heptacosane
3	Docosane	17	Octacosane
4	Tricosane	18	(Z)‐9‐Nonacosene
5	(Z)‐9‐Tetracosene	19	(Z)‐7‐Nonacosene
6	(Z)‐5‐Tetracosene	20	Nonacosane
7	Tetracosane	21	Unknown
8	(Z)‐11‐Pentacosene	22	Hentriacontane
9	(Z)‐9‐Pentacosene	23	24‐Methylene cholesterol
10	(Z)‐7‐Pentacosene	24	Campesterol
11	Pentacosane	25	Dotriacontane
12	Hexacosane	26	Unknown
13	(Z)‐11‐Heptacosene	27	Unknown
14	(Z)‐9‐Heptacosene		

**Figure 7:**
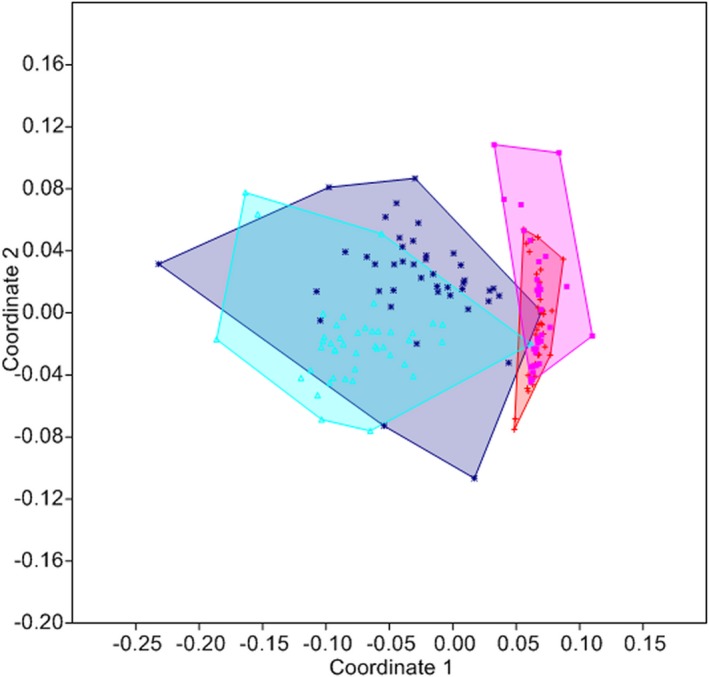
Comparison of the odor bouquets of the antennae of male and female *Osmia bicornis* at different temperature settings (male low temperature (blue; *n* = 43), male high temperature (red; *n* = 34), female low temperature (turquoise; *n* = 44), and female high temperature (pink; *n* = 34); Bray–Curtis similarity measure; stress = 0.2235; subsequent ANOSIM:* R* = 0.6574, *p* < .05 for all pairwise comparisons)

We compared the differences between the two different temperature settings in male and female *Osmia* bees. The results of the NMMDS and the one‐way ANOSIM revealed a significant difference between females and males of *O. bicornis* and also an effect of temperature (*R* = 0.6574, *p* < .05; sequential Bonferroni significance; Figure 7).

The main substances responsible for the separation among the different groups were, according to their importance, Campesterol, (*Z*)‐7‐nonacosene, (*Z*)‐9‐heptacosene, pentacosane, hexacosane, tetracosane, and 24‐methylene cholesterol (SIMPER contribution > 5%). The cumulative contribution of these substances was 53%. Further analysis also revealed that some substances like (*Z*)‐7‐nonacosene are more abundant at higher temperatures, whereas some substances like hexacosane are less abundant. Additionally, according to the boiling points of all substances (PerkinElmer Inc. 2016), these differences are not based solely on physicochemical traits, and the substances with higher boiling points are not necessarily more abundant at higher temperatures (e.g., pentacosane is more abundant at high temperatures whereas both tetracosane and hexacosane are less abundant).

**Figure 8:**
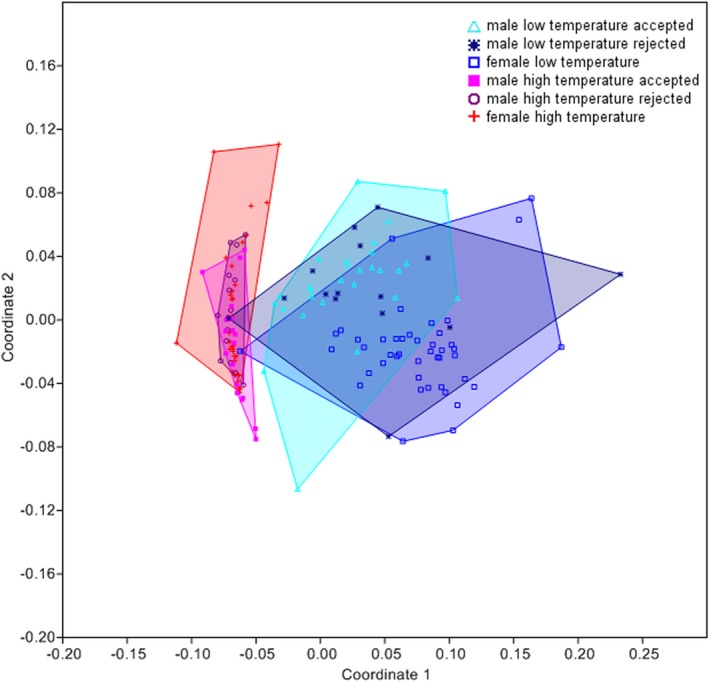
Comparison of the odor bouquets of the antennae of accepted and rejected males versus female *Osmia bicornis* at different temperature settings (male low temperature accepted (*n* = 28), male low temperature rejected (*n* = 15), female low temperature (*n* = 44), male high temperature accepted (*n* = 19), male high temperature rejected (*n* = 15), and female high temperature (*n* = 34); Bray–Curtis similarity measure; stress = 0.2235; subsequent ANOSIM:* R* = 0.6339, *p* < .05 for all pairwise comparisons between temperatures)

A closer look at accepted and rejected males showed that odor profiles differed significantly between different temperatures but not between accepted and rejected males within each temperature setting (Bray–Curtis similarity measure; stress = 0.2235; subsequent ANOSIM: *R* = 0.6339, *p* < .05 for all pairwise comparisons between temperatures; Figure 8).

Furthermore, the odor profiles of males at low temperatures were closer to the females’ odor profiles at low temperatures, and the males at high temperatures were closer to the females at high temperature. Therefore, temperature changes in males and females are similar. The pairwise *R* values revealed that, overall, temperature settings (males *R* = 0.7358; females *R* = 0.8949) affected differences between odor profiles more than sex (low‐temperature *R* = 0.4183; high‐temperature *R* = 0.1420).

## 4 DISCUSSION

Our results clearly show that temperature has a significant effect on both male thorax vibrations and odor bouquets of male antennae. Interestingly, the differences in vibrations were not only found on temporal parameters such as pulse duration, as is the case in many orthoptera (Walker, 1975) and anurans (Gayou, 1984), but temperature also influenced the main frequency and modulation range of the males’ vibrations. Further analyses showed that when groups were separated into accepted versus rejected males, only the vibrations of rejected males were influenced by air temperature and differed in main frequency and pulse duration, whereas the accepted males showed no significant differences. Therefore, knowing that the vibrations play an important role in female choice (Conrad & Ayasse, 2015; Conrad et al., 2010), we conclude that female preference for vibrational signals of males does not change with temperature (Figure 1a). However, males that are not able to show consistency in their vibrations independent of ambient air temperature are not accepted by females. Consequently, males that are less influenced by a change in temperature might have an advantage in mating. They may be able to mate on colder days and much earlier during the day when temperatures are still low while other males that cannot achieve the desired signals are rejected. This would give them a tremendous advantage, as they compete with a smaller number of males for the emerging females. These results differ from previous studies, which showed temperature coupling as is the case in crickets (Pires & Hoy, 1992). However, *Osmia* females do not seem to adapt their preference but instead it is actually the males that need to adapt.

The results from our chemical analysis showed a significant effect of temperature on the odor bouquets of bees. This is not surprising, as numerous studies have shown that odor profiles change with temperature (Collins, Haverty, & Thorne, 1997; Gibbs et al., 1998; Hadley, 1977; Toolson, 1982; Woodrow, Grace, Nelson, & Haverty, 2000). However, it has been hypothesized that those compounds without a signal function, or those species in which the CHC profile does not play a major role in sexual communication, are the ones primarily affected, whereas substances with signal function are long‐chained ones with high boiling points, which are less affected by temperature (Cobb & Jallon, 1990; Noorman & Den Otter, 2002). Contrary to this, we found that the same compounds responsible for the differences between temperature settings are also responsible in separating accepted from rejected males (Conrad et al., 2010) and even separating different populations of *Osmia* in Europe (Conrad, unpublished data). Furthermore, the changes in odor profiles we found cannot be explained by physicochemical traits alone, as compounds with different boiling points change in their quantity and some substances increase whereas others decrease. The compounds differing between the two temperature settings therefore most likely have functions in communication and are not solely used as a water barrier. Therefore, the change in odor bouquet needs to be an active process, as opposed to a solely passive change based on boiling point, at least in part, as some substances are actually increasing at higher temperatures, and some of the compounds have a signal function (Conrad, unpublished), which would be disrupted by a solely passive change through temperature. In lady beetle larvae, it has recently been shown that temperature influences the emissions of infochemicals. This change then leads to a modification in the oviposition behavior of females (Sentis et al., 2015). Compounds with a signal function, which are also affected by temperature, have also been found in the female sex pheromone of *Andrena* bees, which consists of a mixture of alkanes and alkenes. Here, it was shown that alkenes have a key function to attract males (Ayasse, Stökl, & Francke, 2011; Schiestl et al., 1999).

The scope of the differences in odor between the different temperature settings is even more surprising. The differences between male and female odor profiles are actually smaller than the differences we found between the different temperature settings. Usually, we would expect the differences between the sexes to be really distinct as the males use the odor to recognize a female (Rosner, 1994). This means that the impact of temperature on the odor profile is in fact quite large, which might point to either natural or sexual selection driving this differentiation.

A few studies have already shown that temperature changes might also influence the perception of compounds. Linn, Campbell, and Roelofs (1988) found that in Lepidoptera, the male perception of the female pheromone might be altered due to an increase in temperature. At 20°C, these males exhibit a high specificity for a blend of compounds close to the natural pheromone, but at 26°C, the specificity of the males is greatly reduced. The authors believe that this effect is counteracted by preferences for a certain mating temperature, during which the perception is at its optimum. If this was the case in *O. bicornis* as well, it would mean that the range of temperature, during which mating occurs, is not as large as we thought and would only occur at a set temperature of, for example, 18°C. However, as we have observed matings in the field during spring at various different temperatures (personal observation), we believe that temperature fluctuations do play a role.

We therefore expect females to adapt to a change in the male's odor in some way when choosing a suitable partner (Figure 1b). One possible way for the female to do this might be using phenotype matching, during which the female compares her own odor with that of the male. As the females own profile changes with the temperature, this would be a way to adapt her preference accordingly. This matches our results showing that male odor and female odor were more similar at the same temperatures. Further studies, including bioassays, have to be performed in the future to shed a light on these complex interactions.

With current developments regarding climate change, the effects of temperature on the mating of *Osmia* we found are of even greater importance. Taxa adapted to cooler climates often suffer strongly under global warming with longer larval development, smaller larval size, or a loss of suitable habitat, which leads to range retractions (Ott, 2010; Stuhldreher, Hermann, & Fartmann, 2014; Thomas, Franco, & Hill, 2006). In light of our results, it is easily conceivable that mason bees, being adapted to relatively low temperatures in spring time, will be influenced by the increase in temperature that we face—either through disruption in their mate choice, which might lead to a loss of their temperature adaptations, or through having to move to higher latitudes or elevations to avoid the higher temperatures.

Bees in general and especially solitary bees have an important function as pollinators, and their decline or loss could have profound environmental and economic consequences (Vanbergen & the Insect Pollinators Initiative 2013). The global economic value of pollination has been estimated as US$215 billion, and most of that is attributable to insect pollination (Gallai, Salles, Settele, & Vaissière, 2009). Climate change is one of the key pressures on pollinators, and we need more studies like these to estimate the scope of changes we are facing (Andrew et al., 2013; Gallai et al., 2009).

We conclude that temperature strongly influences the male mating signals in *O. bicornis,* and a change in temperature could have a major impact on communication between sexes, female choice, and sexual selection in this species. This might ultimately lead to unforeseeable evolutionary changes in this species. Temperature is therefore an important aspect to consider, not only in future studies on mating behavior, but also in view of climate change and raising temperatures.

## CONFLICT OF INTEREST

The authors declare that they have no conflict of interest.

## AUTHOR CONTRIBUTIONS

T.C. and M.A. involved in the conceptualization. T.C. and M.A. involved in the methodology. C.S. and T.C. involved in the investigation. M.A. contributed to the resources. T.C. wrote the original manuscript. T.C., C.S. and M.A. wrote, reviewed, and edited the manuscript. T.C. and C.S. involved in the visualization.
