# Supplementary material for: The effect of temperature on male mating signals and female choice in the red mason bee, Osmia bicornis (L.)
Source: Ecol Evol. 2017 Sep 23;7(21):8966–75. doi: 10.1002/ece3.3331 (PMC5677480; doi:10.1002/ece3.3331)
Supplement: Supplementary file 1 [file ECE3-7-8966-s001.pdf]

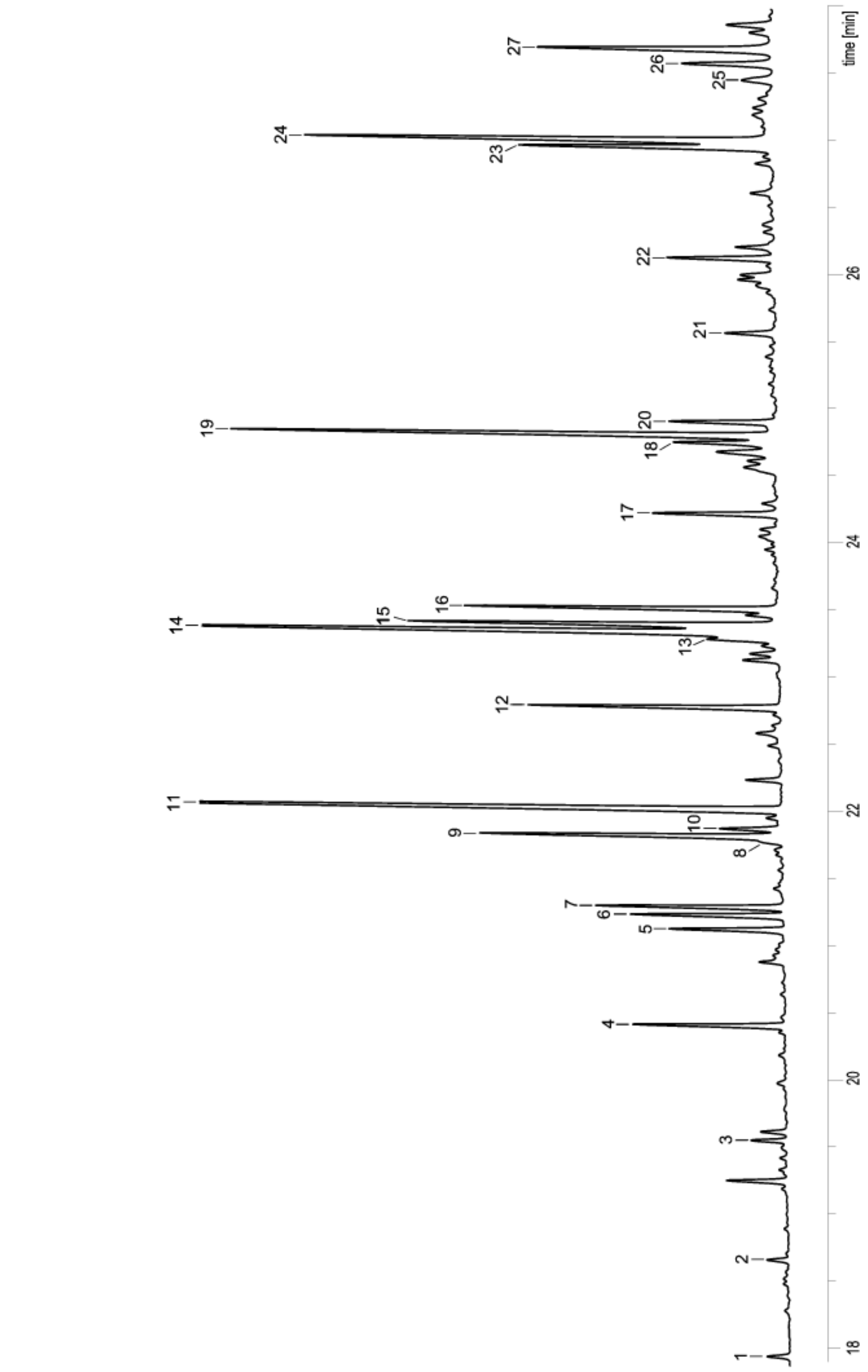

Supplementary Figure 1: Gas chromatogram of a male *O. bicornis* antennal extract. Chemical separations were performed on a non-polar DB-5 mass spectrometry (MS) column and labelled peaks were identified using GC/MS. Numbered peaks correspond to the compounds listed in table 1.
